# The Climate-Health Divide: How Climate Change Will Rewire Health Care Across High-, Middle-, and Low-Income Settings

**DOI:** 10.3390/ijerph23070902

**Published:** 2026-07-14

**Authors:** Francisco Epelde

**Affiliations:** 1 Internal Medicine Department, Parc Taulí Hospital Universitari, Institut d’Investigació i Innovació Parc Taulí (I3PT-CERCA), Universitat Autònoma de Barcelona, 08208 Sabadell, Spain; fepelde@gmail.com; 2 APTIMA Centre Clinic, Mutua de Terrassa, 08221 Terrassa, Spain

**Keywords:** climate change, health systems, global health, adaptation, health equity, planetary health, climate resilience, health-care decarbonisation, low- and middle-income countries

## Abstract

**Highlights:**

**Public health relevance—How does this work relate to a public health issue?**

- Climate change is presented as a major public health issue because it increases climate-sensitive health risks, including heat-related illness, infectious disease shifts, food and water insecurity, mental health impacts, occupational exposure, and disruption of health-care services.
- The work highlights that these risks are unequally distributed: high-income countries face fragile, carbon-intensive health systems, while middle- and low-income countries face heterogeneous but often amplified exposure, weaker surveillance, limited infrastructure, uneven insurance coverage, and constrained fiscal capacity.

**Public health significance—Why is this work of significance to public health?**

- This work is significant because it reframes climate change not only as an environmental threat but also as a structural determinant of health, health-system performance, and health equity.
- It shows that climate resilience and healthcare decarbonisation must be addressed together, since health systems are both vulnerable to climate shocks and contributors to the environmental crisis through emissions, waste, and resource-intensive care.

**Public health implications—What are the key implications or messages for practitioners, policymakers and/or researchers in public health?**

- Practitioners and health systems should integrate climate competence into routine care, including heat-risk assessment, medication review, protection of vulnerable patients, disaster continuity planning, and clinical protocols for heat, smoke, infectious disease, pregnancy, chronic disease, and mental health risks.
- Policymakers, healthcare leaders, donors, local governments, and researchers should prioritise climate-resilient, low-carbon health systems through investments in primary care, surveillance, safe water, cooling, clean energy, cold chains, workforce protection, equitable adaptation, and research that compares the health, cost, equity, and emissions impact of different interventions.

**Abstract:**

Background: Climate change is increasingly recognised not only as an environmental emergency but also as a structural determinant of health and health-system performance. Its clinical consequences will not be distributed evenly: high-income countries face rising heat mortality, infrastructure fragility, ageing-related vulnerability, and the need to decarbonise technologically intensive care; middle- and low-income countries face heterogeneous but often more compressed combinations of heat, infectious disease, food insecurity, water stress, displacement, conflict-related fragility, and limited fiscal capacity. Objective: This structured narrative review proposes a comparative framework for understanding how climate change will transform health care across high-, middle-, and low-income settings and identifies adaptation priorities that are resilient, equitable, and low-carbon. Methods: We synthesised major climate-health assessments, peer-reviewed epidemiological studies, modelling papers, systematic and scoping reviews, and health-system decarbonisation literature identified through targeted searches and reference chaining. Five climate-health pathways, specified a priori from established direct, indirect, and socially mediated pathway frameworks, were used to organise the review. Findings: Climate change will reshape health care through five interacting pathways: direct thermal injury and extreme-weather mortality; altered infectious disease ecology; food, water, and nutritional insecurity; mental, maternal-child, and occupational impacts; and damage to the infrastructure, workforce, supply chains, finances, and emissions profile of health systems. In high-income countries, climate stress exposes the limits of hospital-centred, carbon-intensive, just-in-time care. In middle-income countries, expanding coverage and technology coexist with uneven insurance, large informal workforces, and rapidly growing emissions. In low-income and fragile settings, the same hazards interact with undernutrition, weak surveillance, under-resourced primary care, and constrained finance to produce larger marginal health losses. Conclusions: The central contribution is the concept of the climate-health divide: the unequal conversion of shared climate hazards into clinical demand, service disruption, financial stress, and emissions-intensive responses. Climate resilience and healthcare decarbonisation should therefore be designed together rather than treated as separate agendas.

## 1. Introduction: From Environmental Threat to Health-System Stress Test

Climate change has moved from being a background ecological concern to a central determinant of clinical demand, public-health risk, and health-system solvency. The Sixth Assessment Report of the Intergovernmental Panel on Climate Change concluded that human-induced warming is already producing widespread adverse impacts and that health risks increase with every increment of warming [1,2]. Earlier landmark health commissions framed climate change as potentially the greatest global health threat of the twenty-first century and, conversely, climate action as a major public health opportunity [3,4]. That duality is now unavoidable: the climate crisis is both a disease amplifier and a prevention agenda.

The health literature has matured from describing isolated hazards to analysing pathways through which climate drivers become clinical and health-system risks. Foundational work by McMichael and subsequent climate-health assessments distinguish direct effects, such as heat, floods, and storms; indirect ecosystem-mediated effects, such as air quality, food and water insecurity, and vector change; and socially mediated effects through livelihoods, displacement, conflict, poverty, and health-system capacity [1,2,5,6]. These pathways do not operate independently. They interact with age, occupation, housing quality, baseline disease burden, gender, poverty, migration status, insurance coverage, and the resilience of health services.

A central injustice is that countries and communities least responsible for historic greenhouse gas emissions often experience the largest health consequences and have the least capacity to adapt. Ebi and Hess described this asymmetry as inequity in both causes and consequences [7]. Recent Lancet Countdown reports have reinforced the point: delayed mitigation is already exposing populations to record-breaking climate-health threats, while rapid action would deliver large health co-benefits through cleaner air, healthier diets, active transport, and more resilient systems [8,9,10,11,12]. Income categories are useful but imperfect. This review uses high-income countries (HICs) and low- and middle-income countries (LMICs) as broad shorthand, while explicitly distinguishing middle-income and low-income settings when adaptive capacity, insurance coverage, surveillance, infrastructure, or conflict-related vulnerability differ. For example, insurance coverage varies substantially across low-, lower-middle-, and upper-middle-income countries, and fragile and conflict-affected states face climate risks amplified by conflict, dependence on rain-fed agriculture, and weak institutional capacity [13]. Vulnerability also exists within HICs, and adaptive capacity also exists within poorer countries.

Against this background, this review asks: how do major climate-sensitive health pathways translate into health-system risks across HICs, middle-income countries, and low-income countries, and which adaptation priorities can strengthen resilience while avoiding higher emissions and widening inequity? Its central contribution is the concept of the climate-health divide, defined here as the unequal conversion of shared climate hazards into clinical demand, service disruption, financial stress, and emissions-intensive responses. Operationally, this divide is assessed across four dimensions: exposure and baseline disease burden; social vulnerability and protection; health-system adaptive capacity; and the carbon and resource intensity of care. The review does not seek to rank countries mechanically but to identify how the same hazard creates different system failures and policy needs.

## 2. Methods

This article is a structured narrative review and conceptual synthesis rather than a formal systematic review. The guiding question was: how do climate-related hazards and mitigation choices affect clinical demand, service continuity, infrastructure, workforce protection, finance, equity, and emissions across HICs, middle-income countries, and low-income countries? The population and context were national and subnational health systems; the exposures were climate-related hazards, climate-sensitive ecological changes, and health-care mitigation choices; and the outcomes of interest were health-system vulnerability, adaptation priorities, clinical implications, equity effects, and health-care decarbonisation.

Sources were identified through targeted searches of PubMed/MEDLINE and Google Scholar; review of major climate-health assessments; and reference chaining from IPCC, Lancet Countdown, planetary health, and sustainable health-care publications. Searches combined terms such as climate change, health systems, climate resilience, adaptation, decarbonisation, low-carbon health care, high-income countries, low- and middle-income countries, heat, infectious disease, vector-borne disease, food security, water security, nutrition, pregnancy, child health, mental health, occupational heat, disasters, supply chains, health-care emissions, and universal health coverage. English-language sources published up to 2025 were considered, with emphasis on systematic and scoping reviews, multi-country epidemiological studies, modelling analyses, major international assessments, and policy papers directly relevant to health-system transformation.

Sources were included when they addressed one or more of the following: climate-sensitive health risks with direct implications for health services; differences in exposure, vulnerability, or adaptive capacity across income settings; operational resilience of facilities, workforces, supply chains, or essential services; health-care decarbonisation; or implementation-relevant policy and finance. Sources were excluded when they were single-event commentaries without broader analytical relevance, were not clearly linked to health or health systems, lacked an English abstract or full text, or duplicated evidence already captured by more comprehensive reviews. Because this was a narrative review, no pooled effect estimates or formal risk-of-bias score was calculated.

The five climate-health pathways were specified a priori as organising domains derived from established direct, indirect, and socially mediated climate-health pathway frameworks, including the work of McMichael and subsequent IPCC assessments [1,2,14]. They should therefore be interpreted as design parameters for the review rather than as statistically derived findings. They were selected because each pathway repeatedly appears in climate-health assessments and has direct consequences for health-system operations.

Evidence was synthesised using a framework approach. Findings were first grouped by pathway, then compared across three overlapping dimensions: income context (HIC, middle-income, low-income), health-system function (demand, workforce, infrastructure, supply chains, finance, emissions), and policy horizon. The three policy horizons were immediate clinical preparedness, structural adaptation, and health-system transformation, reflecting the distinction between emergency readiness, resilient service delivery, and low-carbon redesign in the health-system resilience and sustainable-care literature [15,16,17,18]. Table 1 summarises the conceptual framework used to organise the narrative review.

## 3. Narrative Review Findings

The findings are presented according to the a priori pathways in Table 1, followed by cross-cutting implications for mitigation co-benefits and health system operations.

### 3.1. Heat: The Most Immediate Clinical Frontier

Heat is the clearest example of climate change as a direct clinical exposure. Large multi-country analyses have attributed a measurable fraction of heat-related mortality to anthropogenic climate change [19]. Global assessments of deadly heat have warned that combinations of temperature and humidity can exceed human physiological tolerance, especially when exposure is prolonged and cooling is unavailable [20]. Heat affects health through cardiovascular strain, dehydration, renal stress, respiratory exacerbation, sleep loss, impaired cognition, and interactions with medications, including diuretics, anticholinergics, beta-blockers, and psychotropics [21,22].

In high-income countries, heat is already a major mortality risk, particularly in older urban populations. The European summers of 2022 and 2023 demonstrated that wealthy regions with advanced health systems are not immune to climate mortality [23,24]. Urban heat islands, ageing demographics, social isolation, under-cooled housing, and electricity dependence make heat a health-system problem rather than merely a weather problem. Modelling in hundreds of European cities suggests that future heat-related mortality could rise substantially without adaptation, even as cold-related deaths decline [25,26]. At the global level, emerging attribution evidence suggests that climate change is already producing measurable health losses beyond a single hazard, underscoring why heat preparedness should be part of broader health-system adaptation [59]. The novelty for wealthy countries is that heat resilience will require clinical practice, social care, housing policy, labour regulation, and electricity planning to work as a single health intervention.

In LMICs, heat is often more continuous, more occupational, and less medically visible. Outdoor workers, agricultural labourers, street vendors, construction workers, pregnant women, and residents of informal settlements can experience repeated thermal stress without diagnosis or compensation. Middle-income countries may have expanding hospital capacity and insurance schemes, yet large informal workforces and unequal access to cooling can leave many people unprotected. Low-income and fragile settings more often face direct deficits in water, electricity, emergency transport, labour protection, and primary care. Heat-related kidney injury, dehydration, pregnancy complications, and productivity loss may therefore accumulate as a chronic burden rather than as a dramatic emergency spike.

The health-system response to heat should become more ambitious. High-income countries need heat-safe hospitals, home-care registries for isolated older adults, medication review protocols before heat waves, climate-informed emergency staffing, and cooling strategies that do not simply increase emissions. LMICs require occupational heat standards, shaded and cooled primary-care spaces, reliable water access, early-warning systems linked to community health workers, and finance for passive cooling. In both contexts, heat is a test of whether health care can move upstream from rescue to anticipation.

### 3.2. Infectious Diseases: Redistribution Rather than Simple Expansion

Climate change is altering the geography, seasonality, and intensity of infectious disease risks, but the pattern is not a simple global increase everywhere. Temperature, rainfall, humidity, land use, mobility, human behaviour, vector control, immunity, and health-system capacity all shape transmission. A major synthesis reported that more than half of known human pathogenic diseases can be aggravated by climatic hazards [31]. Modelling studies project substantial changes in the suitability for Aedes-borne viruses, including dengue, chikungunya, Zika, and yellow fever [32]. Climate change may also increase opportunities for cross-species viral transmission as wildlife ranges shift [33].

For high-income countries, the infectious disease issue is one of preparedness for unfamiliar risks. Vector-borne diseases may appear in regions where clinicians have little experience, laboratories lack routine testing pathways, and public expectations assume that such diseases are imported rather than locally transmitted. Surveillance systems, wastewater monitoring, genomic epidemiology, travel medicine, and blood-supply safety will become more climate-sensitive. The COVID-19 pandemic also revealed the fragility of public trust, supply chains, and surge capacity; climate change may repeatedly test these same systems through vector, zoonotic, and waterborne events.

For LMICs, climate-sensitive infections overlap with existing burdens of malaria, dengue, diarrhoeal disease, cholera, and neglected tropical diseases. Climate projections for malaria show potential geographic shifts rather than uniform expansion, meaning that some populations may experience new exposure while others see altered seasonality [34]. Middle-income countries may face rapidly urbanising Aedes-borne disease risks alongside uneven insurance coverage and surveillance quality, whereas low-income and conflict-affected settings are more likely to experience simultaneous constraints in sanitation, diagnostics, vaccination, vector control, and outbreak finance. Reviews of infectious disease in an era of global change emphasise that climate interacts with urbanisation, migration, conflict, biodiversity change, and health infrastructure [35,36]. Cholera remains a classic example of climate-water-health interaction, with temperature, rainfall, plankton ecology, and water safety shaping risk [37].

Waterborne disease is especially important for healthcare planning. Systematic reviews link diarrhoeal disease to temperature, rainfall, flooding, and drought, with risk mediated by sanitation, safe water, and behaviour [38]. Food- and water-borne disease burdens are likely to be most severe where infrastructure and surveillance are least robust [39]. The operational implication is that climate adaptation cannot be reduced to hospital preparedness. It requires investment in laboratories, vector control, safe water, sanitation, vaccination, community surveillance, and trusted risk communication.

### 3.3. Food, Water, and Nutrition: The Hidden Clinical Workload

Climate change will affect health through food systems long before many patients present with recognisable “climate disease”. Modelling studies project that future food production under climate change may affect global and regional mortality through changes in diet, body weight, and fruit and vegetable availability [40]. Elevated carbon dioxide can reduce the concentration of protein, zinc, and iron in major crops, threatening human nutrition even when calories are available [41,42]. Climate impacts on food security also include crop failure, price volatility, livestock stress, fisheries disruption, and the loss of livelihoods [43].

In high-income countries, the most visible nutritional effect may not be famine but inequality in diet quality. Food price shocks disproportionately affect low-income households, people with chronic disease, and communities already living in food deserts. Diet-related non-communicable diseases may worsen if healthy foods become less affordable and ultra-processed foods remain cheap. Hospitals and public institutions also face a procurement paradox: they must secure reliable food supply while shifting toward lower-carbon, healthier menus.

In LMICs, climate-food pathways can be more direct and severe, but they are not uniform. Middle-income countries may experience food-price shocks, dietary-quality deterioration, and obesity-undernutrition double burdens, while low-income countries are more exposed to undernutrition, child stunting, unsafe water, and climate-sensitive diarrhoeal disease. Child stunting, micronutrient deficiency, low birth weight, infectious disease susceptibility, and impaired cognitive development are all sensitive to food insecurity. Modelling of child stunting through income and food price pathways illustrates how climate can influence long-term development through economic and nutritional mechanisms [44]. Water scarcity and unsafe water increase diarrhoeal disease, malnutrition, and maternal risk, while floods can contaminate water systems and damage clinics.

Health systems often treat the consequences of food and water insecurity while lacking authority over the causes. A climate-smart health strategy must therefore include nutrition surveillance, school feeding, safe water infrastructure, social protection, climate-resilient agriculture, and procurement reform. The most powerful clinical intervention may be a policy that prevents the patient from becoming malnourished, dehydrated, or infected in the first place.

Table 2 translates the pathway findings into adaptation priorities. The table is not intended as a universal prescription; it shows how similar policy domains require different implementation choices depending on infrastructure, surveillance, insurance coverage, fiscal space, and workforce conditions.

### 3.4. Maternal, Child, Mental, and Occupational Health: Climate Vulnerability Across the Life Course

Climate change is often described through extreme events, but some of its most consequential effects occur through vulnerable life stages and socially patterned exposure. Pregnancy is a critical example. Systematic reviews associate high temperatures with increased risks of preterm birth, low birth weight, and stillbirth [45]. A review focused on the United States also found associations between heat, air pollution, and adverse birth outcomes, demonstrating that wealthy countries share this risk [46]. Children face distinctive vulnerabilities because of developmental physiology, dependency, longer future exposure, and the lifelong consequences of early undernutrition, infection, displacement, and trauma [47].

The contrast between high-income countries and LMICs is again a matter of amplification. In wealthy settings, pregnant people may have access to antenatal care but still live in overheated homes, work in heat-exposed jobs, or experience wildfire smoke. In LMICs, heat and food insecurity may coincide with anaemia, malaria, long travel distances to obstetric care, and under-resourced neonatal services. Climate-safe maternal and child health therefore requires both clinical protocols and structural interventions: cooling, hydration, transport, nutrition, malaria prevention where relevant, and continuity of essential services during disasters.

Mental health is another domain in which climate change will expand healthcare demand while challenging conventional service models. Empirical studies link temperature and climate-related disasters to mental-health risks, including distress, depression, anxiety, post-traumatic stress, and suicide risk [48,49,50,51]. Eco-anxiety and grief may be particularly salient among young people and communities witnessing ecosystem loss. However, mental health impacts are not only psychological reactions to abstract climate futures; they are also responses to material losses-homes burnt, farms flooded, income disrupted, and communities displaced.

Occupational health may become one of the most politically important climate-health domains. Heat reduces labour capacity, especially in outdoor and manual work [27,28]. Multi-model analyses suggest that climate change can affect both labour productivity and labour supply [29]. High-income countries will need enforceable heat standards, worker compensation systems, and redesign of work schedules. LMICs will need the same protections but in labour markets where informal work is common and regulatory reach is limited. Without occupational adaptation, climate change will transfer risk to the bodies of workers least able to refuse exposure.

Floods, storms, and wildfires add acute trauma and chronic displacement to these life-course risks. Floods are associated with mortality, injury, infectious disease, mental-health effects, and disruption of care [52]. In health-system terms, disasters convert social vulnerability into clinical demand and simultaneously damage the infrastructure needed to respond. The future of disaster medicine will therefore be inseparable from housing policy, land-use planning, insurance design, primary care, and community trust.

### 3.5. Air Pollution and Mitigation Co-Benefits: The Fastest Health Dividend

The health argument for climate mitigation is strengthened by the fact that many emissions sources also harm health immediately. Fossil-fuel combustion produces fine particulate matter and other pollutants that contribute to cardiopulmonary disease, cancer, adverse pregnancy outcomes, and premature mortality. Studies estimating the health effects of removing fossil-fuel emissions or reducing fine particulate pollution suggest very large potential public-health gains [60,61,62,63]. This matters politically: the benefits of decarbonisation are not confined to distant climate stabilisation but include cleaner air, fewer hospitalisations, and fewer deaths in the near term.

High-income countries have a special responsibility because their historic emissions and high per-capita energy use have contributed disproportionately to the problem. Their health systems also have substantial carbon footprints, especially through pharmaceuticals, devices, buildings, anaesthetic gases, imaging, laboratories, procurement, and waste. Decarbonisation can therefore be framed as patient safety: a health service that treats asthma while emitting pollution is not fully healing the population it serves.

LMICs face a different but equally important opportunity. Many still need large expansions of energy, transport, housing, sanitation, and healthcare access. If this expansion follows fossil-intensive pathways, future health burdens will increase; if it leapfrogs to clean energy, active mobility, resilient buildings, and efficient care models, the health gains can be enormous. The ethical point is not to limit care in poorer countries, but to finance a pathway in which universal health coverage and low-carbon development reinforce each other.

### 3.6. Health Systems as Victims and Perpetrators of Climate Change

Health systems are harmed by climate change but also contribute to it. Global assessment of the environmental footprint of health care shows that the sector has a material climate and ecological impact [53]. National studies of the National Health Service in England and the United States demonstrate that high-income health systems are energy- and resource-intensive, with emissions embedded in supply chains, buildings, pharmaceuticals, devices, and clinical practice [54,55,56]. This creates a moral and operational contradiction: health care exists to protect life, but its current model can intensify environmental conditions that damage health.

A planetary healthcare framework, therefore, asks health systems to become simultaneously resilient, low-carbon, and equitable [57]. Policy literature is also beginning to translate this lens into health-policy design and resilient-care agendas [15,16], while environmental accounting shows that health-care impacts extend beyond carbon alone [58]. This does not mean rationing necessary care; it means reducing waste, overdiagnosis, low-value interventions, unnecessary travel, inefficient procurement, and avoidable admissions through prevention and better chronic disease management. It also means designing care closer to communities, using telehealth where appropriate, and building facilities that can operate during heat waves, floods, power outages, and supply-chain disruptions.

In high-income countries, decarbonisation is technically feasible but institutionally difficult. Hospitals often reward throughput, technological escalation, and defensive practice. Procurement contracts prioritise cost and reliability over emissions. Clinical guidelines rarely include carbon consequences, and quality metrics seldom reward avoided care. Yet climate-resilient, low-carbon care can align with better medicine: prevention, rational prescribing, antimicrobial stewardship, active transport, plant-forward diets, and reduced low-value care.

In LMICs, the priority is not to copy the most carbon-intensive version of Western medicine. Middle-income countries may face a dual challenge: expanding insurance coverage, specialist care, and hospital technology while preventing wasteful, high-emission care models. Low-income countries require investment in resilient primary care, distributed renewable energy, robust supply chains for essential medicines, oxygen continuity, climate-informed surveillance, and community health workers. Fragile and conflict-affected settings face the greatest implementation constraints because climate exposure, conflict, and weak institutional capacity amplify one another [13]. The challenge is financing. Climate adaptation funds have historically underweighted health, and many health ministries lack the fiscal space to retrofit facilities or build resilient systems. Climate finance should treat health systems as critical infrastructure.

## 4. Discussion

### 4.1. The Climate-Health Divide as Policy Frame

The “climate-health divide” can be understood as the widening gap between populations that can buffer climate hazards through infrastructure, insurance, public services, and political voice, and populations forced to absorb hazards directly through their bodies, income, and caregiving networks. This divide exists between countries, within countries, and within cities. It is not identical to national income, but income strongly shapes the resources available for adaptation.

For high-income countries, the most impactful message may be that adaptation cannot be delegated to emergency departments. Heat plans, flood response, wildfire smoke clinics, and disaster preparedness will fail if housing, labour, transport, social care, and energy systems remain maladapted. Ageing societies will need registries of vulnerable people, home-based care continuity, climate-safe nursing homes, and neighbourhood cooling. Health services should become “anchor institutions” that use their purchasing power, data, and public trust to improve community resilience.

For LMICs, the most impactful message may be that climate adaptation is inseparable from universal health coverage. A health system without reliable electricity, water, workforce protection, cold chains, laboratories, and primary care cannot be climate resilient. Conversely, climate-resilient investments can accelerate universal health coverage if they strengthen everyday services as well as disaster response. Solar-powered clinics, safe water systems, community surveillance, and resilient supply chains are climate interventions and health-system interventions at the same time.

Across both contexts, adaptation must avoid a trap: protecting the already protected while leaving the most exposed behind. Cooling centres that require transport may miss isolated older adults. Digital alerts may miss people without smartphones. Insurance-based recovery may exclude informal workers. Hospital flood barriers may protect tertiary care while primary clinics fail. The ethical test of climate-health policy is therefore distributional: who benefits first, who is missed, and who pays.

The review findings also identify a set of implementation-orientated research and policy questions. Table 3 summarises these questions and links them to equity safeguards so that adaptation and decarbonisation are evaluated by who benefits, who is missed, and whether health-system resilience is strengthened without increasing emissions or inequity.

### 4.2. Clinical Implications

Clinicians will increasingly need climate competence. This does not mean that every clinician becomes a climate scientist. It means recognising climate-sensitive risk in ordinary practice: an older patient on diuretics during a heatwave; a pregnant worker exposed to extreme temperatures; a child with asthma during wildfire smoke; a patient with diabetes whose insulin supply depends on electricity; a person with severe mental illness living in overheated housing; a dialysis patient during a flood; or a migrant family exposed to unfamiliar vectors.

Clinical records and public-health systems should begin to encode climate-relevant vulnerability without stigmatising patients. Heat risk, housing insecurity, electricity dependence, mobility limitations, occupational exposure, pregnancy, renal disease, cardiopulmonary disease, and medication profiles can inform proactive outreach. However, data alone are insufficient. Registries must be linked to home visits, medication review, cooling support, transport, and emergency planning.

Medical education should treat climate change as a core topic in epidemiology, pathophysiology, ethics, and health systems. The curriculum should avoid apocalyptic abstraction and instead teach practical competence: recognising heat illness, advising patients during smoke events, protecting maternal and child health, maintaining care during disasters, reducing waste in clinical practice, and advocating for policies that prevent disease.

### 4.3. Governance and Finance

The governance of climate and health remains fragmented. Environment ministries may own emissions policy; health ministries may own clinical services; finance ministries may control adaptation budgets; local governments may control housing, transport, and land use. Yet climate-health risks cross these boundaries. A heat wave is a meteorological event, a housing event, an electricity event, a labour event, and a health event. Governance should therefore include formal climate-health units with authority to coordinate surveillance, adaptation planning, facility standards, procurement, and emergency response.

Finance is the decisive constraint, especially for LMICs. The most credible climate-health agenda will translate clinical risks into investable projects: resilient primary-care facilities, solar power and batteries, safe water, oxygen continuity, cold chains, early-warning systems, laboratories, and workforce protection. In high-income countries, finance should be redirected from reactive disaster spending and low-value care toward prevention, resilient infrastructure, and decarbonisation. In both settings, procurement can become a climate-health lever by demanding lower-carbon supply chains and essential-service continuity.

Measurement also needs reform. Health systems commonly measure mortality, admissions, bed occupancy, and cost, but rarely measure avoided climate harm, emissions per care pathway, facility resilience, or the distribution of adaptation benefits. A health system can appear efficient on a normal day and fail catastrophically under climate stress. Future performance metrics should therefore include climate readiness, carbon intensity, service continuity during extremes, and equity of protection.

### 4.4. Policy Implementation: Responsible Actors

Implementation requires assigning responsibility rather than treating climate-health action as a general aspiration. Ministries of health should establish climate-health units, set facility resilience standards, integrate climate risk into surveillance and emergency planning, and align universal health coverage with climate adaptation. Hospitals and healthcare networks should stress-test power, water, cooling, digital systems, supply chains, waste, and clinical surge capacity; they should also reduce low-value care and use procurement to demand lower-carbon, reliable supply chains. Primary care systems should maintain registries of heat-, smoke-, electricity-, and mobility-vulnerable patients; link early warnings to outreach; and protect essential medicines, vaccines, oxygen, dialysis, maternity care, and chronic disease services during shocks.

Local governments should treat housing, cooling, transport, urban greening, air-quality shelters, safe water, and land-use planning as health interventions. Donors, development banks, and climate-finance mechanisms should fund resilient primary care facilities; solar power and batteries; cold chains; laboratories; workforce protection; and safe water systems, especially in low-income and fragile settings. Professional societies and universities should incorporate climate competence into clinical guidelines, accreditation, and continuing education. Researchers should evaluate implementation packages using health, cost, equity, service-continuity, and emissions metrics rather than single-hazard outcomes alone.

### 4.5. Compound Events and Invisible Failures

The next decade of climate and health will be defined less by single hazards than by compound events. A heat wave that coincides with wildfire smoke, power failure, staff shortages, and high bed occupancy is different from a heat wave in isolation. A flood that closes roads during a dengue outbreak is different from a flood in a setting with spare beds, functioning laboratories, and reliable ambulances. Compound events expose an uncomfortable truth: health-system resilience is not the sum of separate emergency plans. It is the ability to maintain essential services when several assumptions fail at the same time.

High-income countries often have sophisticated hospitals but fragile dependencies. Electronic records, imaging, intensive care, dialysis, operating theatres, sterilisation units, blood banks, and pharmaceutical refrigeration all require electricity, water, transport, digital systems, and supply chains. Climate hazards can interrupt any of these inputs. This means that the future climate vulnerability of a wealthy hospital may be hidden not in its clinical expertise but in its loading dock, procurement software, generator fuel, cooling system, and workforce housing. A hospital can be clinically excellent and operationally brittle.

In low-resource settings, the invisible failures are different but equally dangerous. A rural clinic may not formally “collapse”; it may simply run out of staff, fuel, clean water, rapid tests, oxytocin, oxygen, insulin, vaccines, or referral transport. Patients may arrive too late because roads are flooded, crops have failed, or household income has disappeared. These failures are often undercounted because deaths occur outside hospitals or because routine health-information systems degrade during crises. Climate change therefore threatens not only health outcomes but also the ability to see and measure those outcomes.

This is why climate-health policy should move beyond facility-by-facility emergency preparedness toward network resilience. A resilient health system is not one flagship hospital behind a flood wall; it is a distributed network of primary care, emergency transport, laboratories, pharmacies, community workers, data systems, safe water, electricity, and social support. The network must be designed so that when one node fails, others can maintain essential functions. This approach is especially important for maternal care, dialysis, insulin-dependent diabetes, HIV and tuberculosis treatment, vaccination, cancer therapy, and mental-health continuity. This networked view is consistent with contemporary reconceptualisations of health-system resilience as a property of everyday system functioning, not only emergency response [17].

### 4.6. Toward a Climate-Resilient Model of Care

A climate-resilient model of care would look different from the dominant twentieth-century model. It would shift emphasis from late rescue to early prevention, from centralised fragility to distributed continuity, from carbon-intensive throughput to value and resilience, and from disease-specific silos to community protection. In practice, this means more investment in primary care, public health, home-based support, social prescribing, nutrition, vaccination, mental-health integration, and chronic disease control. These interventions are not glamorous, but they reduce the number of people who become acutely vulnerable during climate shocks.

For high-income countries, one of the most disruptive implications is that some forms of high-technology medicine may need to be judged by their opportunity costs and environmental consequences. Low-value imaging, unnecessary procedures, avoidable admissions, pharmaceutical waste, and carbon-intensive procurement consume resources that could strengthen resilience. The goal is not less care, but better care: clinically appropriate, lower-carbon, less wasteful, and more preventive. In this sense, climate action can become a quality-improvement agenda rather than a separate environmental programme.

For LMICs, climate-resilient care should not be interpreted as an instruction to remain low-resource. The objective is universal access to safe, modern care without reproducing the most wasteful aspects of high-income systems. Solar-powered clinics, efficient oxygen systems, essential diagnostics, digital decision support, resilient supply chains, and community health workers can expand access while reducing vulnerability. International financing should support this leapfrogging rather than forcing countries to choose between development and decarbonisation.

Research should also become more decision-orientated. Many climate-health studies estimate risk, but fewer compare the health, cost, equity, and emissions consequences of specific adaptations. Health leaders need to know which combinations of cooling, outreach, facility retrofit, clean energy, surveillance, workforce protection, and social protection save the most lives under plausible local scenarios. The next generation of climate-health science should therefore integrate epidemiology, implementation research, economics, engineering, behavioural science, and ethics. Climate-smart public-health frameworks make the same point by linking surveillance, adaptation, mitigation, and equity into a single resilience agenda [60,61,62,63].

This framing is intended to make the manuscript’s contribution more analytical than descriptive. Climate change is not an external environmental chapter added to medicine; it is a force that is rewriting the operating conditions of medicine itself. The climate-health divide links health-system vulnerability, inequality, and decarbonisation into one policy problem, and it is therefore relevant to both wealthy and low-resource settings.

## 5. Limitations

This manuscript is a narrative synthesis and does not provide a formal systematic review, pooled effect estimates, or a new quantitative model. The search strategy was structured but not exhaustive, and the selection of sources involved interpretive judgement. Climate-health evidence is heterogeneous, and risks vary substantially by geography, time horizon, socioeconomic context, baseline disease burden, governance, conflict, and adaptation. Some impacts are well quantified, such as heat-related mortality, while others, such as health-system disruption, mental health, displacement, and compound events, remain harder to measure. The distinction between HICs, middle-income countries, and low-income countries is useful for policy comparison but can obscure vulnerability within wealthy countries and resilience within poorer ones.

The article also emphasises health systems, which means that some ecological determinants, biodiversity changes, and non-health-sector adaptation policies are discussed only insofar as they affect health care. Finally, the future will depend strongly on mitigation. A world of rapid emissions reduction and equitable adaptation will produce very different health outcomes from a world of delayed action, high inequality, and fragile governance.

## 6. Conclusions

Climate change will affect health care in every country, but not in the same way. HICs will confront rising climate-sensitive demand in ageing, expensive, technologically dependent, and carbon-intensive systems. Middle-income countries will face a mixed agenda of expanding coverage, uneven insurance, urban infectious risks, informal labour exposure, and growing health-care emissions. Low-income and fragile settings will confront a denser convergence of heat, infection, food and water insecurity, displacement, occupational exposure, weak infrastructure, and constrained fiscal capacity. The shared challenge is that climate change turns weaknesses in health systems into clinical harm.

The most impactful response is not simply more emergency capacity. It is a transformation of health systems into institutions that prevent climate-sensitive disease, remain functional during shocks, reduce their own emissions, and protect the people who are most exposed. The future of health care will be judged not only by what happens inside hospitals but by whether societies can keep people safe enough that they do not need rescue in the first place.

## Figures and Tables

**Table 1:** Climate-sensitive health pathways and expected contrast across high-, middle-, and low-income settings.

Pathway	Core Mechanisms	High-Income Countries	Middle- and Low-Income Settings
Heat and thermal extremes	Heat illness, dehydration, renal/cardiopulmonary strain, medication interactions, and sleep loss [19,20,21,22].	Older adults, social isolation, poorly cooled housing, grid and cooling dependence [23,24,25,26].	Outdoor/informal work, limited cooling, water, emergency care and insurance; pregnancy and kidney risks [19,20,21,22,27,28,29,30].
Infectious disease ecology	Vector shifts, altered seasonality, spillover, water- and food-borne disease [31,32,33,34,35,36,37,38,39].	Emerging vector risks put pressure on surveillance, labs, blood safety and travel medicine [31,32,33,34,35,36].	Higher burden where sanitation, vaccination, diagnostics and outbreak finance are constrained; conflict may amplify risk [13,34,35,36,37,38,39].
Food, water and nutrition	Crop instability, micronutrient dilution, water scarcity, diarrhoeal disease and price shocks [40,41,42,43,44].	Food-price and diet-quality impacts, especially for low-income households [40,41,42,43,44].	Undernutrition, stunting, unsafe water and climate-sensitive diarrhoeal disease, especially in low-income settings [40,41,42,43,44].
Mental health and social disruption	Trauma, distress, depression, grief, displacement and livelihood loss [27,28,29,45,46,47,48,49,50,51,52].	Recognised but uneven mental-health access after wildfire, flood and heat disasters [46,48,49,50,51,52].	Needs are often underdiagnosed and underfunded; weak social protection increases chronic distress [13,27,28,29,45,47,48,49,50,51,52].
Health-system operations	Facility damage, supply-chain interruption, power/water failure, workforce heat, fiscal shock and emissions [15,16,53,54,55,56,57,58].	Energy dependence, just-in-time logistics and ageing infrastructure: decarbonisation is urgent [15,16,53,54,55,56,57,58].	Essential services can fail quickly; resilience depends on primary care, surveillance and infrastructure finance [13,16,17,18,30].

**Table 2:** Differential adaptation priorities across income settings.

Policy Domain	High-Income Countries	Middle- and Low-Income Settings	Common Principle
Heat-health protection	Heat maps, social care outreach, medication review, heat-safe hospitals, and grid resilience [19,20,21,22,23,24,25,26].	Warnings, water, shaded work, passive cooling, occupational protection and cooled primary care [19,20,21,22,27,28,29].	Protect exposed groups before EDs are overwhelmed.
Infectious disease preparedness	Climate surveillance, emerging-vector diagnostics, labs, public trust and blood/travel protocols [31,32,33,34,35,36].	Vector control, vaccination, sanitation, rapid diagnostics, community workers and outbreak finance [34,35,36,37,38,39].	Surveillance must be linked to action.
Food, water, nutrition	Healthy low-carbon procurement, food-price protection and diet-related disease surveillance [40,41,42,43,44].	Safe water, sanitation, nutrition programmes, resilient crops, social protection and growth monitoring [40,41,42,43,44].	Prevent nutritional and infectious cascades.
Facility resilience	Hospital retrofits, backup power, flood protection, supply-chain redundancy and digital continuity [15,16,53,54,55,56,57,58].	Resilient primary care, solar energy, oxygen, cold chains, roads and communication [13,16,17,18,30].	Essential services must continue during shocks.
Healthcare decarbonisation	Scope 1–3 reduction, procurement reform, pathway redesign and low-value care reduction [15,16,53,54,55,56,57,58].	Efficient resilient infrastructure while expanding access and avoiding high-emission models [16,18,57].	Design mitigation and adaptation together.

**Table 3:** High-impact research and policy questions for the next decade.

Question	Why It Matters	Preferred Evidence or Action	Equity Safeguard
How much demand can systems absorb before quality declines?	Planning often counts hazards but not service failure or workforce attrition [16,17].	Stress-test hospitals and primary care under heat, flood, smoke and supply-chain scenarios.	Stratify by age, income, disability, migration status and geography.
Which adaptations give the greatest health gain per carbon and cost?	Some adaptations may raise emissions or mainly benefit wealthy groups [15,16,18,57,58].	Compare cooling, clean energy, outreach and retrofits using health, cost and emissions metrics.	Prioritise exposure reduction among vulnerable groups.
How should guidelines include climate risk?	Medication safety, pregnancy, chronic disease and mental health are climate-sensitive [21,22,45,46,47,48,49,50,51].	Develop heat/smoke protocols, climate-aware prescribing and continuity plans.	Do not shift responsibility to patients lacking cooling, housing or transport.
How can finance strengthen health systems?	Health adaptation remains underfunded, especially in low-income and fragile settings [13,16,18].	Create bankable projects: resilient clinics, cold chains, water, surveillance and workforce protection.	Require community participation and transparent allocation.
How can decarbonisation improve care?	Poorly designed decarbonisation may be perceived as austerity [15,16,53,54,55,56,57,58].	Target waste, low-value care, procurement, energy, anaesthetic gases and prevention.	Protect essential care and expand access in underserved areas.

## Data Availability

No new data were created or analyzed in this study. Data sharing is not applicable to this article.
